# Incorporating softmax in psychophysical detection models for normal and electric hearing

**DOI:** 10.1016/j.mex.2026.103807

**Published:** 2026-01-28

**Authors:** Savine S.M. Martens, Jeroen J. Briaire, Johan H.M. Frijns

**Affiliations:** a Department of Otorhinolaryngology of Leiden University Medical Center, Leiden, , the Netherlands; b Leiden Institute for Brain and Cognition, Leiden, Bioelectronics Group, EEMCS, Delft University of Technology, the Netherlands

**Keywords:** Cochlear implants, Computational auditory model, Psychometric curve, Psychophysical tests, Softmax function

## Abstract

Modeling psychophysical auditory detection has proven to be difficult, as with existing neural models and detection models, we were unable to adjust the slope of the psychometric curve accurately. In machine learning, the softmax function is an excellent tool to assign probabilities to model outputs. Incorporating this function into psychophysical detection models can enhance the precision of the auditory detection model. This study extended Hamacher’s detection model by integrating a softmax function, providing additional control over the slope of psychometric curves.

• Using computational simulations of both normal and electric hearing, we applied this enhanced model to two psychophysical tasks: masker-probe detection and amplitude modulation detection.
• The outcomes demonstrated that the normal hearing model aligned closely with expected performance, with predictable shifts in psychometric curves as the noise and slope parameters varied. In addition, with the electric hearing model, the new detection model could now reach lower asymptotes in the psychometric curve than with Hamacher’s detection model.
• These findings suggest that incorporating the softmax function provides a flexible tool for modeling auditory psychophysical data. This tool has potential applications for in silico evaluation of speech coding strategies for cochlear implants.

## Graphical abstract

**Figure:**
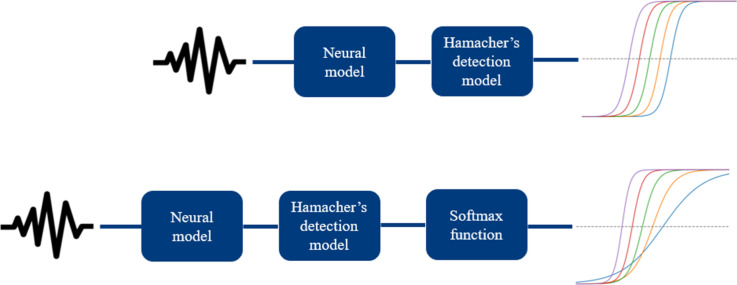

**Specifications table**

**Table:** 

**Subject area**	Neuroscience
**More specific subject area**	*Modelling of auditory psychophysics*
**Name of your method**	*Softmax detection model*
**Name and reference of original method**	*V. Hamacher, "Signalverarbeitungsmodelle des elektrisch stimulierten Gehors (Ph. D. thesis)," RWTH Aachen, Wissenschaftsverlag Mainz, Aachen, Germany, 2004.*
**Resource availability**	*Available upon request*

## Background

Psychophysics examines the relationship between physical stimuli and the sensations they evoke, often represented by psychometric curves that show how the probability of detecting a stimulus changes with stimulus properties (e.g., pitch, intensity) [1,2]. In auditory research, sensory threshold detection is commonly measured using a 3-alternative forced-choice (3AFC) paradigm. Two common hearing paradigms are masker-probe detection, which studies how preceding sounds mask the detection of subsequent ones, and amplitude modulation detection, which evaluates sensitivity to changes in a sound signal's intensity envelope.

The introduction of cochlear implants (CIs) made psychophysics valuable for comparing normal hearing with hearing through electrical stimulation and for tracking improvements in CI users. However, collecting reliable psychometric curves is time-intensive, requiring many repetitions to estimate detection probabilities, particularly when comparing different CI settings, such as improved speech coding strategies. To streamline this process, computational modeling can be used to simulate and assess outcomes before running actual human experiments [3].

Various neural models of cochlear activity based on animal data have successfully simulated the peripheral response [[4], [5], [6], [7], [8]]. However, modeling how these responses translate into human behavior in psychophysical tests remains abstract and complex. Some models combine peripheral cochlear simulations with detection models and incorporate internal noise to mimic neural variability [[9], [10], [11]]. Adjusting this internal noise can shift the psychometric curve horizontally; however, with these models, we were unable to adjust the slope of the psychometric curve.

In the field of artificial intelligence (AI), neural networks offer an alternative, capable of abstracting behavioral insights from neural spikes due to their ability to model complex relationships [12,13]. One of the challenges neural networks face is translating outputs into meaningful probabilities that account for uncertainty [14,15]. A widely used solution is the softmax function, which converts raw model outputs into a probability distribution where the probabilities sum to one [[14], [15], [16]]. The softmax function originates from the Boltzmann distribution, initially used to describe the probability of particles occupying different energy states in thermal equilibrium [17]. It has since been adopted in machine learning and decision theory for interpreting model outputs involving multiple options.

In cognitive modeling, the softmax function is not a novel implementation but rather a well-established approach for linking latent stimulus evidence to observed choice probabilities [18,19]. It is mathematically equivalent to Luce’s choice rule [20] and the multinomial logistic model [21], both of which have long been used to describe perceptual and decision-making behavior. The softmax function has, for example, been used to model human behavior in (neuro)economic decision-making tasks [[22], [23], [24]]. In signal detection theory, the softmax function enables flexible modeling of response probabilities, making it particularly useful for psychophysical applications. Similarly, a multinomial logistic function (equivalent to the softmax function) has been applied to model psychometric curves describing the behavior of monkeys in motion-discrimination tasks [25] and decision tasks [[26], [27], [28], [29]]. In multi-armed bandit tasks, softmax models have been incorporated into signal detection or decision models to predict human behavior [30,31]. Moreover, softmax-based models have been explicitly linked with signal detection theory (SDT), showing that the softmax function can approximate or extend signal detection theory formulations when modeling response probabilities in a visual memory task in humans [32].

Alternative decision rules exist, such as deterministic winner-take-all mappings (choosing the largest internal response, ‘argmax’), fixed threshold/criterion rules [2], or latent variable accumulators such as drift-diffusion and race models [[33], [34], [35]]. However, these alternatives have specific limitations for the present application. For example, simple threshold rules and many internal-noise SDT models primarily shift the psychometric function horizontally with changes in sensory noise (altering threshold), and do not provide a direct, continuous control over slope without modifying model structure [36,37]. In contrast, softmax includes a temperature parameter that acts as a principled decision noise term, tunable independently of threshold and capable of adjusting the slope of the predicted psychometric function, a key requirement for modeling of 3AFC performance across different paradigms. Moreover, latent variable accumulator models are primarily intended for reaction-time paradigms, in which both choice and response time distributions are modeled jointly; because the present study focuses on choice probabilities in detection-based 3AFC tasks without reaction-time measurements, these models would introduce unnecessary complexity without providing additional explanatory benefit. Furthermore, softmax yields smooth, differentiable choice probabilities that readily integrate with likelihood-based parameter estimation and modern computational model fitting, avoiding task-specific heuristics that would otherwise be needed to map model scores to choice probabilities.

Therefore, the softmax function proves to be a useful tool in decision models with classical psychometric functions, providing greater flexibility for describing both the slope and asymptotic behavior of psychometric curves, and may also benefit auditory neural models. In this study, we extended Hamacher's [11] detection model by incorporating the softmax function, aiming to enhance the model’s flexibility, particularly in adjusting the slope of the psychometric curve. This function assigns probabilities to the model outcomes, allowing us to predict behavior in 3AFC tests. We applied this extended model to two psychophysical tasks: amplitude modulation detection and masker-probe detection, comparing the results between normal-hearing participants and CI-stimulated cochleae. This approach offers a more adaptable computational framework for modeling auditory behavior and could reduce participant burden in experimental studies while supporting the evaluation of new CI processing strategies.

## Method details

We updated Hamacher’s 3AFC detection model [11], which includes an 'odd one out' paradigm, where the listener must distinguish the test stimulus, including the reference and the test signal (*RT*), from a stimulus with only the reference (*R*), with several additions (see Appendix for an English summary of the original method). In this detection model, the stimuli are processed to create neural internal representations of the stimuli. Noise is added to these internal representations, which are then compared with an auditory memory.

Firstly, in this study, we utilized two phenomenological models of the auditory nerve with stochastic properties to simulate auditory responses: a normal hearing (NH) model of Bruce et al. [4] and our electric hearing (EH) model for cochlear implants [[6], [7], [8],^38^,^39^]. The normal hearing model simulates the auditory nerve's response to acoustic stimuli, capturing the tonotopic organization of the cochlea. Loudness was set to 65 dB root mean square (RMS) for the reference signal, and then all test signals were matched accordingly. The electric hearing model includes a speech processor, a research version of the Fidelity 120 strategy (‘SpecRes’), and an implanted cochlea model, simulating the auditory nerve response to electric stimuli delivered by a cochlear implant [[6], [7], [8],^38^,^39^]. Loudness in this model was scaled at 65 dB sound pressure level (SPL) following the implementation in [5]. For a more detailed overview of the specific implementation and parameters used in both neural models, we refer to the Materials and Methods, Table 1, and Table A1 in [3]. These models allow us to compare sensory input processing in acoustic and electric hearing across different psychophysical tests, incorporating variability in neural responses. However, any auditory neural model that produces single-fiber spike trains in response to an auditory stimulus can be used.

**Fig. 1:**
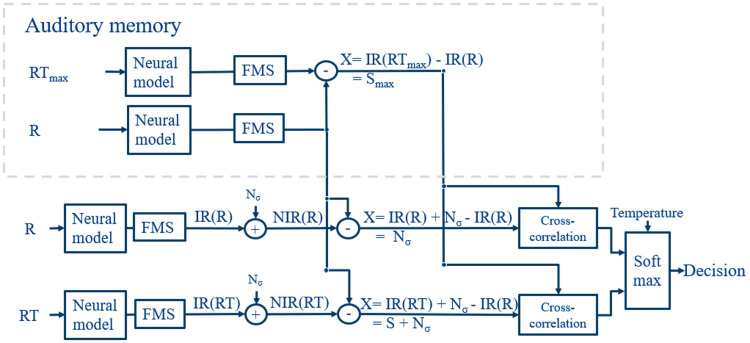
Updated detection model. The auditory memory *S*_*max*_ with the greatest stimulus intensity *RT*_*max*_ is cross-correlated with the noisy internal representations *NIR*, after subtraction of the clean internal representation of the reference *IR*(*R*). These values are used to determine, with the softmax function, the probability of the odd-one-out. *N*_*σ*_ = perceptual noise. *R* = reference stimulus. *RT* = Reference test stimulus. FMS = forward masking stage (see Appendix).

Secondly, we implemented an auditory memory with the greatest stimulus intensity and replaced the max detector of Hamacher’s model with a softmax function to evaluate the probability of detecting the odd one out (see Fig. 1). Following the approach of Hamacher [11], internal representations (*IR*s) were generated from single-fiber responses of the neural models. These analysis representations are correlated with the auditory memory *S*_*max*_, for which the internal representation of the reference *IR*(*R*) is subtracted from the internal representation of the stimulus with the greatest stimulus intensity *IR*(*RT*_*max*_):

(1) $$\begin{array}{c} X=\mathrm{IR}\left(RT_{\max}\right)-\mathrm{IR}\left(R\right)=S_{\max} \end{array}$$

**Fig. A6:**
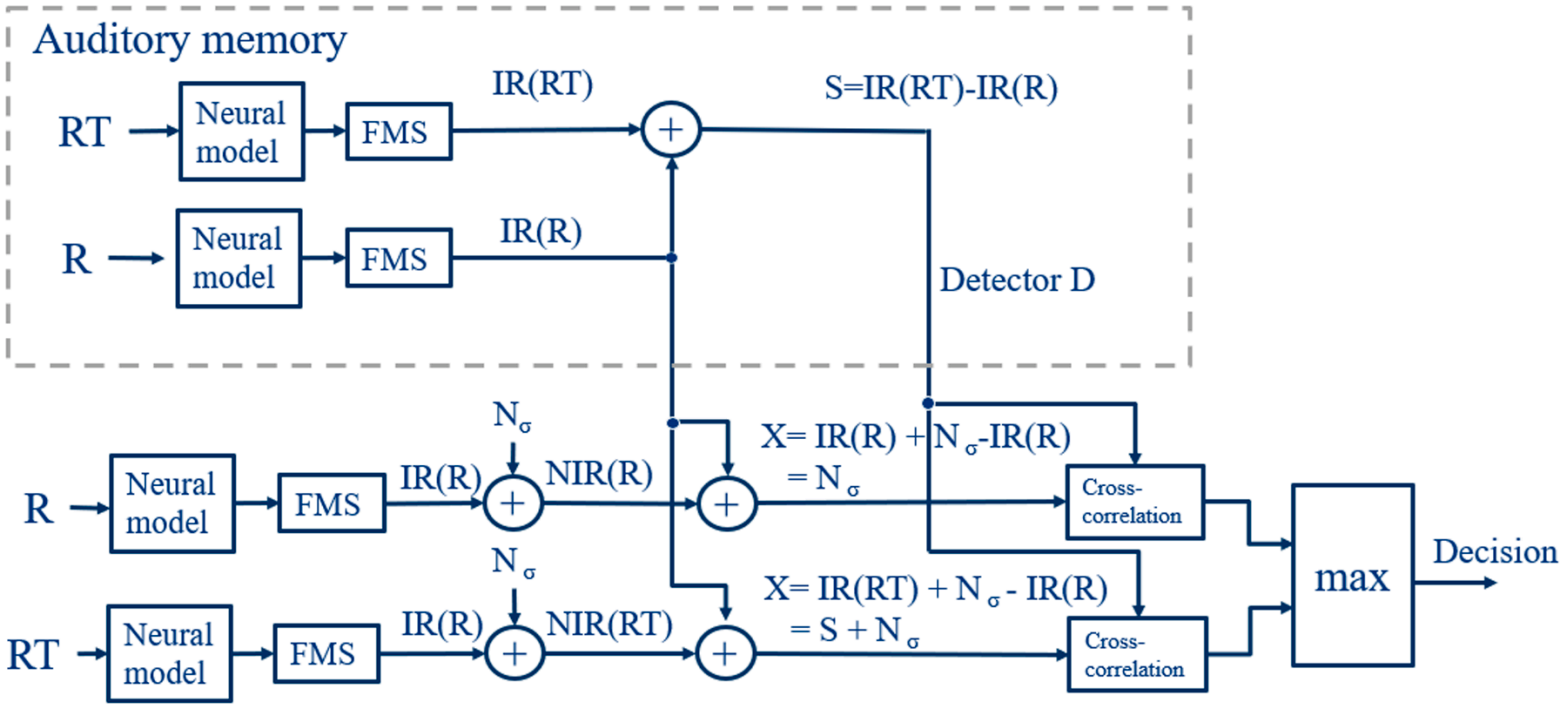
The original detection model of Hamacher [11]. The auditory memory is used to create a detector *D* to which the noisy internal representations *NIR* are correlated, after subtraction of the clean internal representation of the reference *IR*(*R*). The greatest correlation indicates the reference test stimulus *RT*. FMS = forward masking stage. *N*_*σ*_ = perceptual noise.

This is different from the original detection model (see Appendix, Fig. A6 and Eq. (A9)), where, for each trial, the presented *IR*(*RT*) is used to calculate *S*. The *RT*_*max*_ would be, for instance, the stimulus with the greatest amplitude modulation depth or the greatest probe amplitude. We assume this is a more accurate representation of the participant's auditory memory during a psychophysical test, as it is the stimulus presented during the test trial to understand the paradigm.

Thirdly, to allow comparison between the two types of hearing, a relative value was used for noise *N*_*σ*_, because the normal hearing model includes a greater number of fibers in its processing at each characteristic frequency (50 fibers) versus 1 fiber in the electric hearing model. Since we wanted to remain true to the default settings of the models, we chose to implement a relative noise parameter for a more informative and fair comparison between the models. The absolute value of the noise is not critical for comparing different models or stimuli, as it primarily serves to reduce the chance of correct answers by introducing uncertainty. In this case, the standard deviation $\sigma_{w}$ of the Gaussian noise *N*_*σ*_ was scaled:

(2) $$\begin{array}{c} \sigma_{w}=\;\sigma x \end{array}$$

where x is the standard deviation of the *IR*(*R*), per neural model and paradigm. This $\sigma_{w}$ is then used as in the original version (see Eq. (A6)).

Lastly, in the original detection model, the presentation with the highest correlation was selected as the decision for that critical band. In this newly proposed version, the correlations are used to derive a decision by transforming them into a probability with the softmax function. The softmax function with a temperature parameter *T*, a variant introduced in [40], is used as follows,

(3) $$\begin{array}{c} \mathrm{soft}\max\left(z_{i}\right)=\frac{e^{z_{i}/T}}{\sum_{j}e^{z_{j/T}}} \end{array}$$

to provide a probability for the single score *z*_*i*_ based on all raw scores of the input vector *z*. The temperature *T* controls the distribution's smoothness or sharpness. By adjusting the temperature, one can influence how probabilities are distributed among the possible outcomes, in this case adjusting the slope of the resulting psychometric function. An advantage of using this softmax function is that the values of *z* can come from various statistical routines. This results in a probability of *IR*(*RT*) per critical band. The average over all critical bands is taken to derive a probability of the trial. This is repeated over 100 trials, and the average is taken again over these trials.

Thus, to summarize, four changes have been added to the original detection model:

1. Two different and more elaborate neural models have been included.
2. We implemented a memory internal representation that is more representative of the practice trial during psychophysical testing, since this trial is presented most often to the participant and is used to explain the paradigm.
3. A scaling factor is used to enable comparison of the noise variance between the two neural models.
4. The decision is now based on the mean probability calculated with a softmax function instead of the correlation-based maximum.

## Method validation

**Fig. 2:**
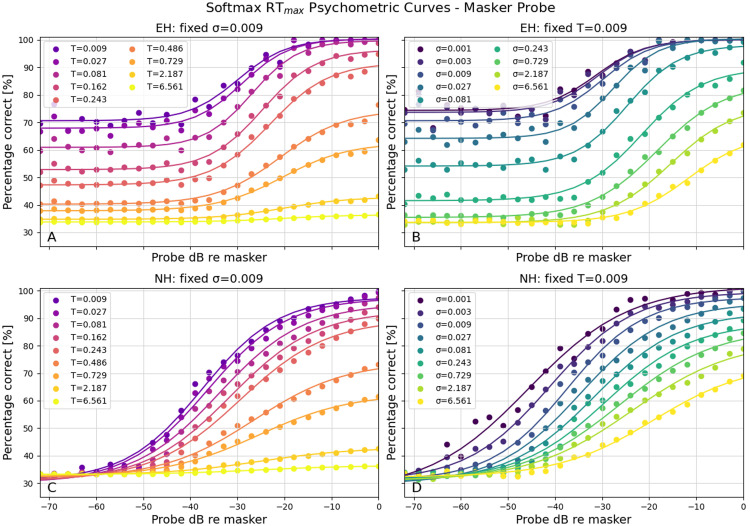
The psychometric curves with the masker-probe paradigm. A) The performance with a fixed σ and varying temperatures for EH. B) The performance with a fixed temperature and varying σ for EH. C) The performance with a fixed σ and varying temperatures for NH. D) The performance with a fixed temperature and varying σ for NH.

The first paradigm on which we tested this was the same masker-probe paradigm as used by Hamacher [11], with a 100-ms 1 kHz masker and after 100 ms of silence, a 10-ms probe of the same frequency, which could vary in amplitude. In Fig. 2, the results of the masker-probe experiment are shown. The top row (A, B) displays the outcomes for the electric hearing model, while the bottom row (C, D) shows those for the normal hearing model. As expected, the normal hearing model demonstrated a clear shift in the psychometric curve with increasing levels of σ, and the slope of the curve (and the maximum value reached) was modified when the temperature parameter changed.

In the electric hearing model (top row), the curves show greater variability with these sets of values for temperature and σ, while they have a higher plateau on the left side of the curve. In both types of hearing, we see greater flexibility in the slope and the position of the psychometric curve.

**Fig. 3:**
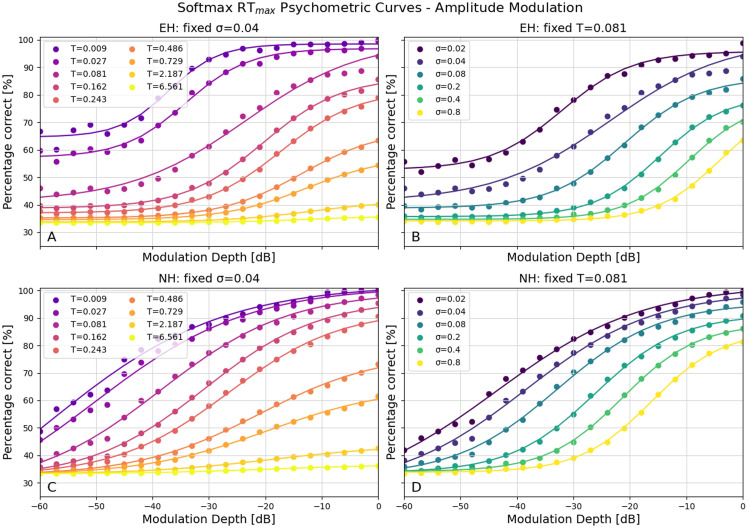
The psychometric curves with the amplitude-modulation paradigm. A) The performance with a fixed σ and varying temperatures for EH. B) The performance with a fixed temperature and varying σ for EH. C) The performance with a fixed σ and varying temperatures for NH. D) The performance with a fixed temperature and varying σ for NH.

**Fig. A7:**
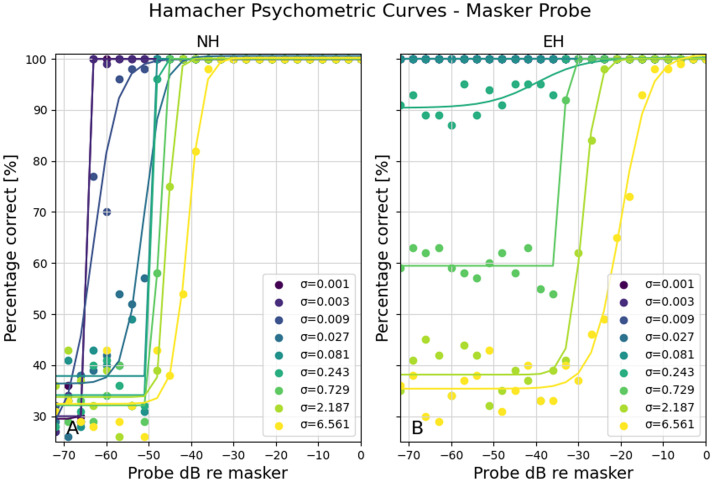
The psychometric curves with the original Hamacher’s detection model with the masker-probe paradigm, for A) normal hearing and B) electric hearing.

**Fig. A8:**
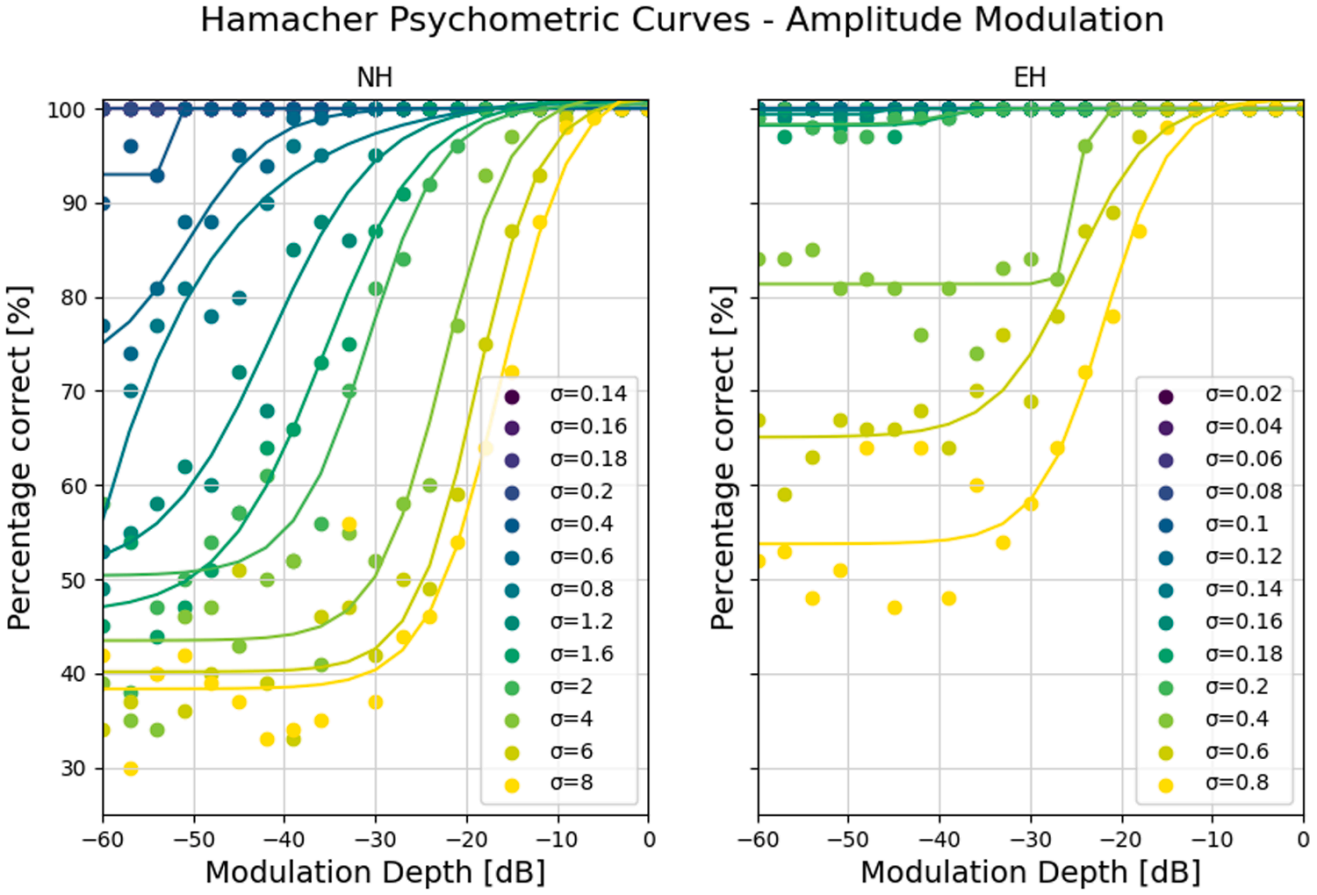
The psychometric curves with the original Hamacher’s detection model with the amplitude-modulation paradigm, for A) normal hearing and B) electric hearing.

The second paradigm was an amplitude modulation paradigm as used in [41]. The odd-one-out consisted of an amplitude-modulated signal (with a carrier frequency of 1500 Hz and a modulation frequency of 40 Hz) versus a pure tone stimulus with the same carrier frequency and maximum amplitude. The modulation depth determined the stimulus intensity. A similar pattern was observed in the amplitude modulation experiment (Fig. 3). For the normal hearing model (Figs. 3C and 3D), the σ and temperature produced the expected outcomes, with a shift in the curve corresponding to increased σ levels and a slope change with varying temperatures. The electric hearing model also showed the same behavior as in the previous paradigm, with a tendency to plateau above chance level and variability in the slope of the curve. Again, there is greater variability in the position and slope of the psychometric curve, allowing for modeling with greater diversity in performance when compared with the previous version of the detection model (see Fig. A7, Fig. A8).

**Fig. 4:**
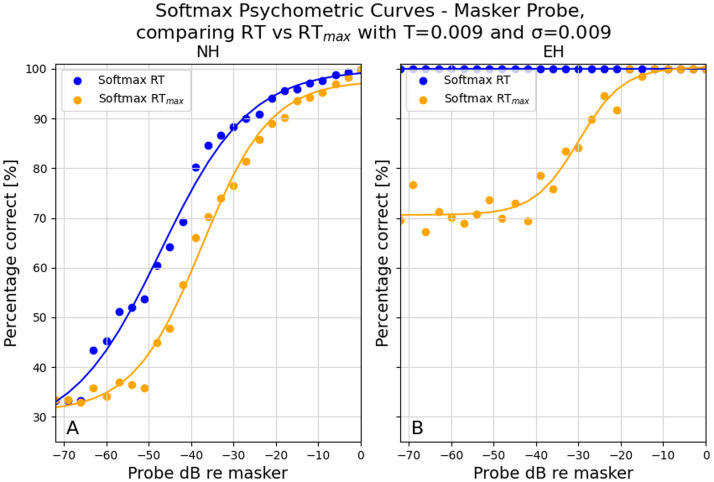
Comparison between using the greatest stimulus (*RT*_*max*_) versus the stimulus per trial (*RT*) as memory in the masker-probe paradigm, created with σ=0.009 and *T* = 0.0009. A) Normal hearing. B) Electric hearing.

Fig. 4 shows the effect of changing the memory representation from the *RT* (current trial) to *RT*_*max*_ (stimulus with the greatest stimulus intensity) for both normal hearing and electric hearing. As a result, the left side of the curves decreases, whereas the right side stays mostly unaffected. This effect is greater for electric hearing than for normal hearing. This represents an improvement in electric hearing, particularly when compared to the previous results using the Hamacher detection model (see Fig. A7, Fig. A8).

## Limitation

This new addition to the detection model provides more control over the psychometric curve; however, it is highly dependent on the neural model that is used. The new implementation can slightly overcome the limitation of plateauing above chance level in comparison with the previous version (see Fig. A7, Fig. A8), as the left side of the curve more closely approximates the chance level with electric hearing (Fig. 4). However, this is at the cost of not reaching 100% correct performance due to the difference in decision-making. In Hamacher’s detection model, the maximum correlation of all critical bands determines which stimulus is chosen, and the percentage is achieved by iterating over the 100 trials. Here, an average is used over the derived probabilities from each critical band, and then again, an average is taken over 100 trials. As a result, critical bands without information (where the stimulus is not present) tend to lower the score.

## Ethics statements

No humans or animals were involved.

## CRediT author statement

**S.S.M. Martens**: Conceptualization, Methodology, Software, Validation, Formal analysis, Investigation, Data Curation, Writing – original Draft, Visualization, Project administration

**J.J. Briaire**: Conceptualization, Writing - Review & Editing, Supervision, Funding acquisition

**J.H.M. Frijns**: Writing - Review & Editing, Supervision, Funding acquisition

## Declaration of competing interest

The authors declare that they have no known competing financial interests or personal relationships that could have appeared to influence the work reported in this paper.

## Figures and Tables

**Fig. A1:**
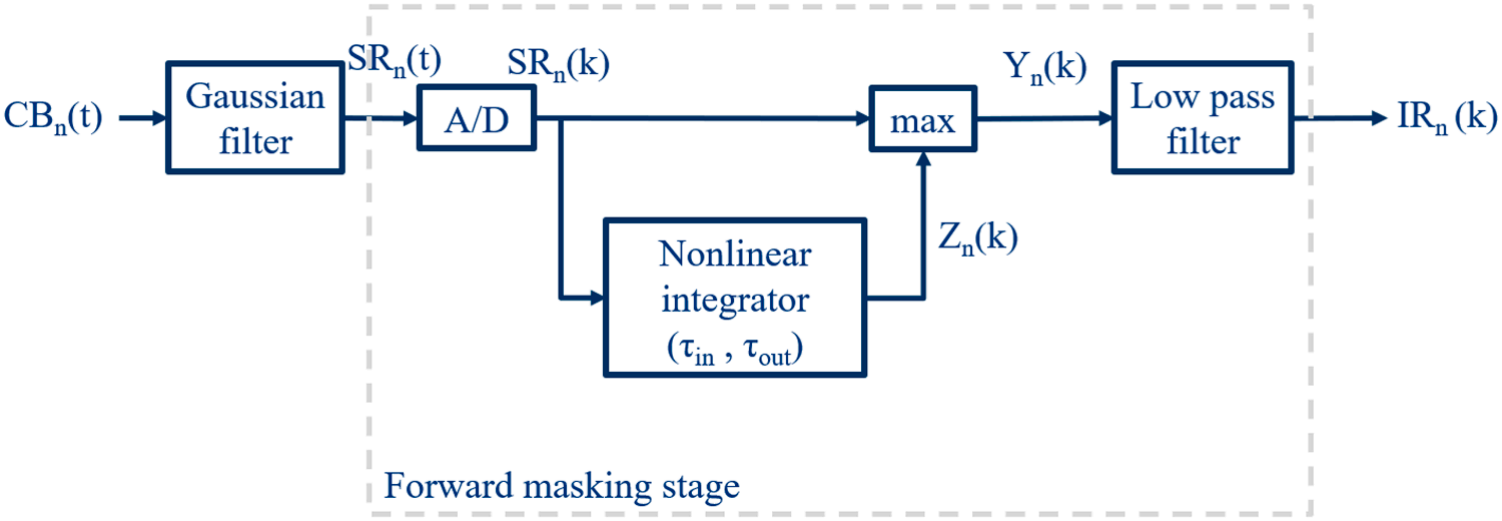
The forward masking stage takes the summed activity over all single fibers in a critical band *n* (*CB*_*n*_) and transforms it into an internal representation *IR* of that critical band *n. SR* = summed response. *Z*_*n*_=perceptual threshold. *Y*_*n*_=state of critical band.

**Fig. A2:**
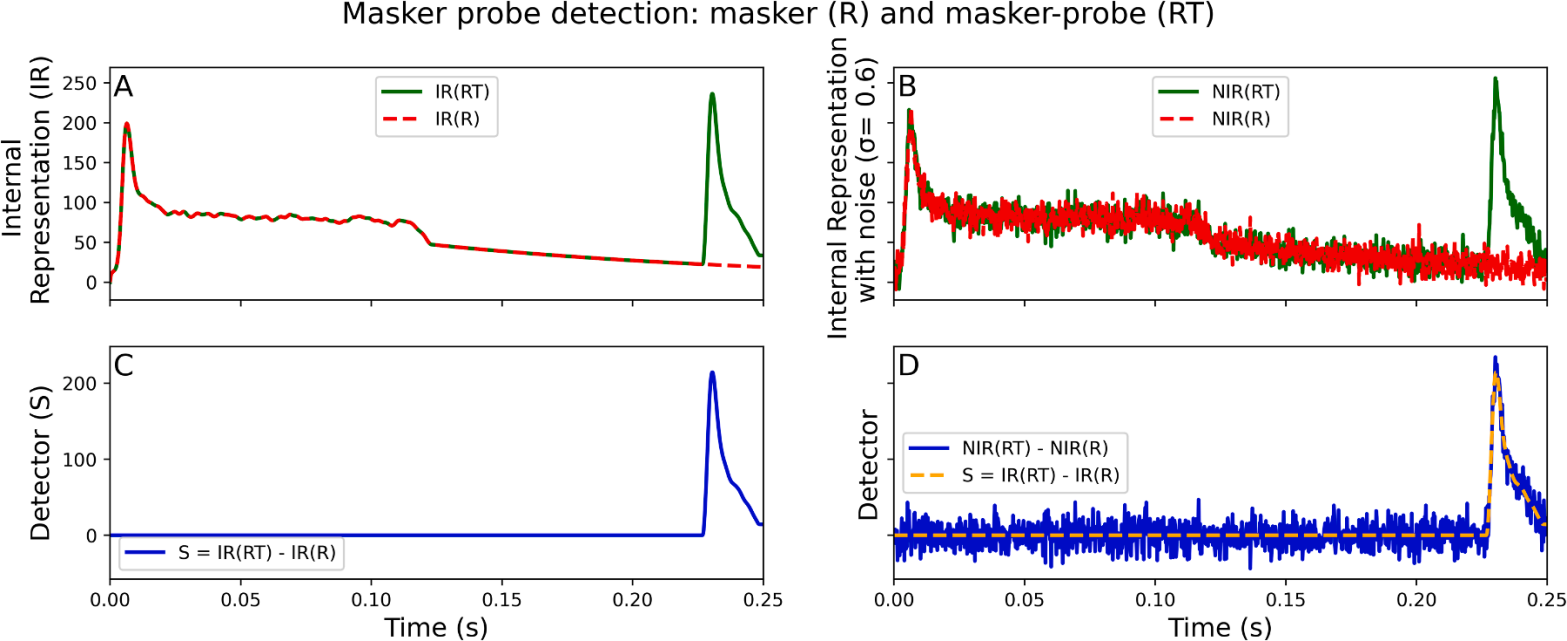
The internal representations of A) probe *IR*(*RT*) and masker *IR*(*R*) with normal hearing. B) The internal representations with added noise created with σ=0.6. C) The resulting analysis representation by subtracting *IR*(*R*) from *IR*(*RT*). D) The subtracted internal representations with and without noise.

**Fig. A3:**
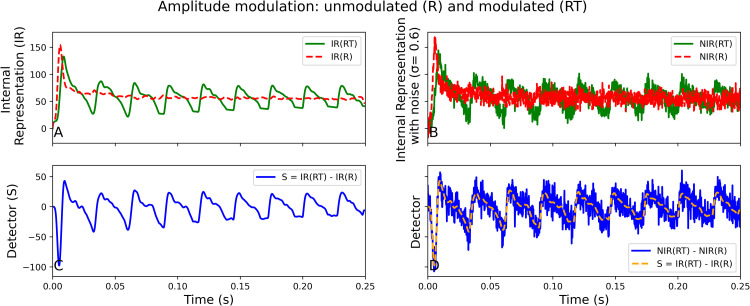
The internal representations of A) modulated signal *IR*(*RT*) and unmodulated signal *IR*(*R*) with normal hearing. B) The internal representations with added noise created with σ=0.6. C) the resulting analysis representation by subtracting *IR*(*R*) from *IR*(*RT*). D) The subtracted internal representations with and without noise.

**Fig. A4:**
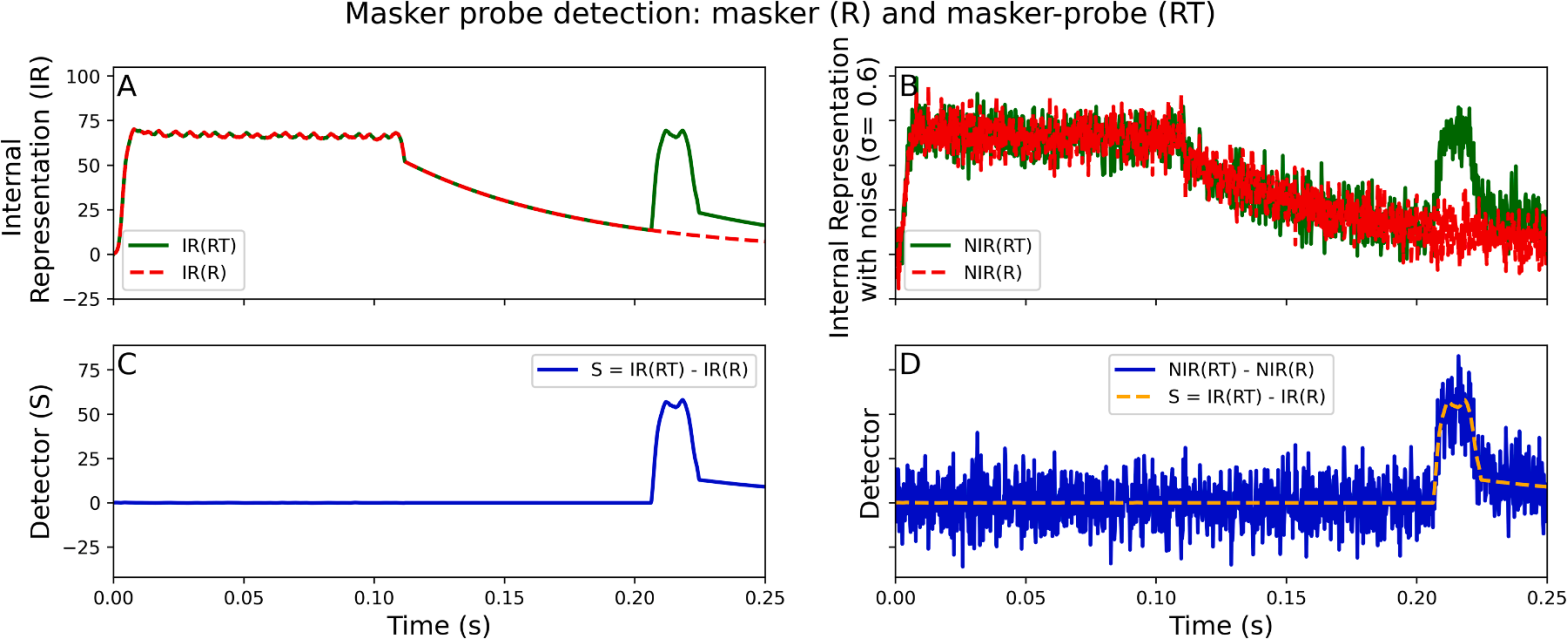
The internal representations of A) probe *IR*(*RT*) and masker *IR*(*R*) with electric hearing. B) The internal representations with added noise created with σ=0.6. C) the resulting analysis representation by subtracting *IR*(*R*) from *IR*(*RT*). D) The subtracted internal representations with and without noise.

**Fig. A5:**
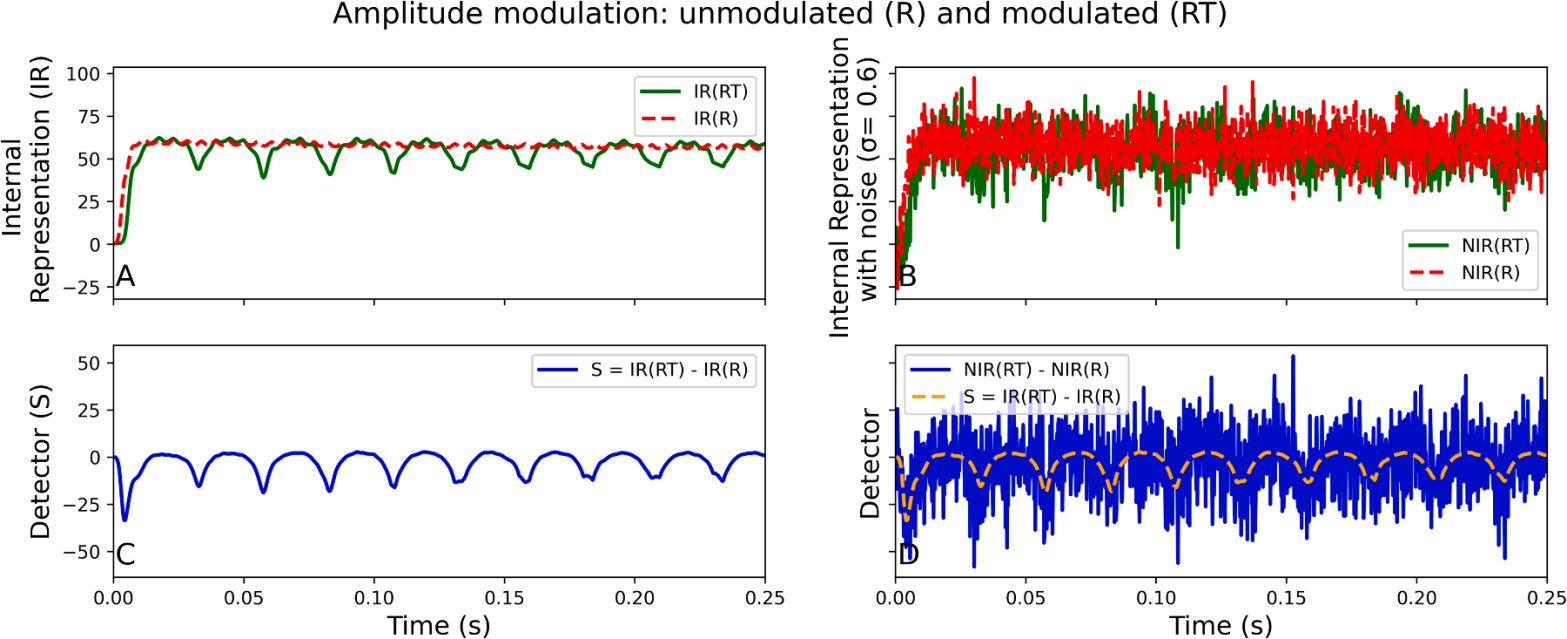
The internal representations of A) modulated signal *IR*(*RT*) and unmodulated signal *IR*(*R*) with electric hearing. B) The internal representations with added noise created with σ=0.6. C) the resulting analysis representation by subtracting *IR*(*R*) from *IR*(*RT*). D) The subtracted internal representations with and without noise.

**Table A1:** Parameters and their values for the forward masking stage (FMS). These parameters are unaltered in our version of the detection model.

Hamacher parameters	Value
$\tau_{G}$	0.001 s
*f*_*s*_	5000 Hz
$\tau_{\text{in}}$	0.07 s
$\tau_{\text{out}}$	0.07 s
*f*_*c*_	500 Hz

## Data Availability

Data will be made available on request.

## Appendix

### Hamacher’s detection model

This method is described in [11]; however, this work is not available online and in English. Therefore, it has been paraphrased here. This detection model (see Fig. A6) creates neural internal representations to distinguish the reference signal *R* from the reference test signal *RT*. The internal representations *IR* are created using the forward masking stage (FMS). The model makes use of assumed auditory memory to compare with noisy internal representations to detect the odd-one-out.

A neural model is used to produce spike trains in response to presented stimuli *R* and *RT*. All spike trains in a critical band are combined to create an internal representation *IR*, which captures the overall neural activity at this critical band level, representing the auditory system’s higher-level processing of stimuli (see Fig. A1). This model incorporates 42 critical bands.

First, the spikes of all fibers within each critical band *CB*_*n*_ are summed, and a Gaussian convolution is applied to obtain a general summed response *SR*_*n*_ of the critical band:

(A1) $$\begin{array}{c} SR_{n}\left(t\right)=CB_{n}\left(t\right)\frac{1}{\sqrt{2\pi }\tau_{G}}\exp\left(-\frac{t^{2}}{2\tau_{G}^{2}}\right), \end{array}$$

with $\tau_{G}$ set to 1 ms. These summed responses *SR*_*n*_ are sampled at a rate of *f*_*s*_ = 5 kHz and then transformed into a state *Y*_*n*_ with perceptual threshold *Z*_*n*_ with:

(A2) $$Y_{n}\left(k\right)=\max\left(SR_{n}\left(k\right),Z_{n}\left(k\right)\right)$$

(A3) $$\begin{array}{c} Z_{n}\left(k\right)=c_{1}\left(k\right)Z_{n}\left(k-1\right)+c_{2}\left(k\right)SR_{n}\left(k\right), \end{array}$$

(A4) $$\begin{array}{c} \text{with}\;c_{1}\left(k\right)=\exp\left(-\frac{1}{\tau_{\text{in}}f_{s}}\right),c_{2}\left(k\right)=1-c_{1}\left(k\right)\;\text{for}\;SR_{n}\left(k\right)\geq Z_{n}\left(k-1\right), \end{array}$$

(A5) $$\begin{array}{c} c_{2}\left(k\right)=\exp\left(-\frac{1}{\tau_{\text{out}}f_{s}}\right),\;c_{2}\left(k\right)=0\;\text{for}\;SR_{n}\left(k\right)<Z_{n}\left(k-1\right), \end{array}$$

with $\tau_{\text{in}}$ and $\tau_{\text{out}}$ are both set to 70 ms. Afterwards, *Y*_*n*_ is low-pass filtered (cutoff frequency: *f*_*c*_ = 500 Hz) to provide an internal representation *IR*_*n*_ of critical band *n*. An overview of all parameters in the FMS is provided in Table A1. The next step is the detection model, which compares the internal representations of the presented stimuli with the detector *D*. It is assumed that there is a clean auditory memory of both *R* and *RT*. The internal representation of the memory of the reference *IR*(*R*) is subtracted from the *IR*(*RT*) to calculate the detector (see Figs. A2, A3, A4, A5).

Perceptual noise *N*_*σ*_ was added to introduce stochasticity into these internal representations. This noise was normally distributed, with an average of zero and a standard deviation $\sigma_{w}$:

(A6) $$\begin{array}{c} N_{\sigma }=\;N\left(0,\;\sigma_{w}\right) \end{array}$$

From each presented stimulus, the clean *IR*(*R*) was subtracted to derive the analysis representation *X*:

(A7) $$\begin{array}{c} X=\mathrm{NIR}\left(R\right)-\mathrm{IR}\left(R\right)=\mathrm{IR}\left(R\right)+N_{\sigma }-\mathrm{IR}\left(R\right)=N_{\sigma } \end{array}$$

(A8) $$\begin{array}{c} X=\mathrm{NIR}\left(\mathrm{RT}\right)-\mathrm{IR}\left(R\right)=\mathrm{IR}\left(\mathrm{RT}\right)+N_{\sigma }-\mathrm{IR}\left(R\right)\;=S+N_{\sigma } \end{array}$$

This analysis representation *X* is correlated to the detector *D*, which is the difference between *IR*(*RT*) and *IR*(*R*):

(A9) $$\begin{array}{c} S=\mathrm{IR}\left(\mathrm{RT}\right)-\mathrm{IR}\left(R\right)=D \end{array}$$

The stimulus producing the largest correlation is assumed to be the odd one out. See Fig. A6 for an overview of the full detection model of Hamacher, with the original auditory memory *S* using the current trial, and a different selection for the greatest odds. The presentation with the greatest number of maximum correlations over all critical bands is chosen as the odd-one-out for that trial. This is repeated over 100 trials to derive a probability.

Fig. A7 shows the resulting psychometric curves with Hamacher’s detection model in a masker-probe paradigm. For normal hearing, with increasing σ, the psychometric curve shifts to the left; however, the slope of the curve is extremely steep. For electric hearing, the curve also steeply shifts to the left, but in addition, the left side of the curve has a different plateau per σ. Fig. A8 shows the resulting psychometric curves with Hamacher’s detection model in an amplitude-modulation paradigm. Normal hearing again shows a consistent slope with increasing σ, although it is less steep compared with the previous paradigm. Electric hearing shows the same behavior as in the previous paradigm: a plateau on the left side above chance level. The σ value allows for little control over the psychometric curve.

## References

[1] Levitt H. (1971). Transformed up-down methods in psychoacoustics. J. Acoust. Soc. Am..

[2] Green D.M., Swets J.A. (1966).

[3] Martens S.S., Briaire J.J., Frijns J.H. (2025). Spectral ripples in normal and electric hearing models. Technologies.

[4] Bruce I.C., Erfani Y., Zilany M.S. (2018). A phenomenological model of the synapse between the inner hair cell and auditory nerve: implications of limited neurotransmitter release sites. Hear. Res..

[5] Venema I.M., Martens S.S., Kalkman R.K., Briaire J.J., Frijns J.H. (2025). Neural correlates of loudness coding in two types of cochlear implants—a model study. Technologies.

[6] Kalkman R.K., Briaire J.J., Dekker D.M., Frijns J.H. (2022). The relation between polarity sensitivity and neural degeneration in a computational model of cochlear implant stimulation. Hear. Res..

[7] van Gendt M.J., Briaire J.J., Kalkman R.K., Frijns J.H. (2016). A fast, stochastic, and adaptive model of auditory nerve responses to cochlear implant stimulation. Hear. Res..

[8] Kalkman R.K., Briaire J.J., Frijns J.H. (2015). Current focussing in cochlear implants: an analysis of neural recruitment in a computational model. Hear. Res..

[9] Dau T. (1999).

[10] Fredelake S., Hohmann V. (2012). Factors affecting predicted speech intelligibility with cochlear implants in an auditory model for electrical stimulation. Hear. Res..

[11] V. Hamacher, "Signalverarbeitungsmodelle des elektrisch stimulierten Gehors (Ph. D. thesis)," *RWTH Aachen, Wissenschaftsverlag Mainz, Aachen, Germany,* 2004.

[12] Saddler M.R., Gonzalez R., McDermott J.H. (2021). Deep neural network models reveal interplay of peripheral coding and stimulus statistics in pitch perception. Nat. Commun..

[13] Brochier T. (2022). From microphone to phoneme: an end-to-end computational neural model for predicting speech perception with cochlear implants. IEEE Trans. Biomed. Eng..

[14] Gawlikowski J. (2023). A survey of uncertainty in deep neural networks. Artif. Intell. Rev..

[15] Hinton G. (2012). Deep neural networks for acoustic modeling in speech recognition: the shared views of four research groups. IEEE Signal Process. Mag..

[16] Guo C., Pleiss G., Sun Y., Weinberger K.Q. (2017). International conference on machine learning.

[17] L. Boltzmann, *Weitere studien über das wärmegleichgewicht unter gasmolekülen*. Aus der kk Hot-und Staatsdruckerei, 1872.

[18] Franke M., Degen J. (2023). The Softmax function: properties, motivation, and interpretation. https://alpslab.stanford.edu/papers/FrankeDegen_submitted.pdf,2023.

[19] van Putten W.L. (1982). Maximum likelihood estimation for Luce's choice model. J. Math. Psychol..

[20] Luce R.D. (1959).

[21] D. McFadden, "Conditional logit analysis of qualitative choice behavior," 1972.

[22] Lopez-Persem A., Rigoux L., Bourgeois-Gironde S., Daunizeau J., Pessiglione M. (2017). Choose, rate or squeeze: comparison of economic value functions elicited by different behavioral tasks. PLoS Comput. Biol..

[23] Cerreia-Vioglio S., Maccheroni F., Marinacci M., Rustichini A. (2023). Multinomial logit processes and preference discovery: inside and outside the black box. Rev. Econ. Stud..

[24] Train K.E. (2009).

[25] Bak J.H., Pillow J.W. (2018). Adaptive stimulus selection for multi-alternative psychometric functions with lapses. J. Vis..

[26] Padoa-Schioppa C. (2022). Logistic analysis of choice data: a primer. Neuron.

[27] Kim S., Hwang J., Lee D. (2008). Prefrontal coding of temporally discounted values during intertemporal choice. Neuron.

[28] Amemori K.-i., Graybiel A.M. (2012). Localized microstimulation of primate pregenual cingulate cortex induces negative decision-making. Nat. Neurosci..

[29] Ferrari-Toniolo S., Bujold P.M., Grabenhorst F., Báez-Mendoza R., Schultz W. (2021). Nonhuman primates satisfy utility maximization in compliance with the continuity axiom of expected utility theory. J. Neurosci..

[30] Reverdy P., Leonard N.E. (2016). Parameter estimation in softmax decision-making models with linear objective functions. IEEE Trans. Auto. Sci. Eng..

[31] Sherratt T.N., O'Neill E. (2023). Signal detection models as contextual bandits. R. Soc. Open Sci..

[32] M.M. Robinson, I. Destefano, T. Brady, and E. Vul, "Revisiting the connection between Luce’s Choice Axiom and Signal Detection theory: application to visual memory," 2022.

[33] Ratcliff R. (1978). A theory of memory retrieval. Psychol. Rev..

[34] Vickers D. (1970). Evidence for an accumulator model of psychophysical discrimination. Ergonomics.

[35] Usher M., McClelland J.L. (2001). The time course of perceptual choice: the leaky, competing accumulator model. Psychol. Rev..

[36] García-Pérez M.A., Alcalá-Quintana R., Woods R.L., Peli E. (2011). Psychometric functions for detection and discrimination with and without flankers. Atten., Percept., Psychophys..

[37] F. Kingdom and N. Prins, "Psychophysics: a practical introduction," 2010.

[38] Nogueira W., Litvak L., Edler B., Ostermann J., Büchner A. (2009). Signal processing strategies for cochlear implants using current steering. EURASIP J. Adv. Signal Process..

[39] de Nobel J., Martens S.S., Briaire J.J., Bäck T.H., Kononova A.V., Frijns J.H. (2024). Biophysics-inspired spike rate adaptation for computationally efficient phenomenological nerve modeling. Hear. Res..

[40] Asadi K., Littman M.L. (2017). International Conference on Machine Learning.

[41] Marrufo-Pérez M.I., Eustaquio-Martín A., López-Bascuas L.E., Lopez-Poveda E.A. (2018). Temporal effects on monaural amplitude-modulation sensitivity in ipsilateral, contralateral and bilateral noise. J. Assoc. Res. Otolaryngol..

